# Understanding the influence and acceptability of health and wellbeing coaching in underserved populations: a qualitative systematic review

**DOI:** 10.1186/s12889-026-28440-8

**Published:** 2026-08-05

**Authors:** Ian Litchfield, Christine Burt, N. Gardner, O. Hart, H. Lea, C. Harewood, J. Naisby, I. Natrins, N. Adams-Quakenbush, N. Thomas, S. Wilson, Zhiyi Zao

**Affiliations:** 1 University of Birmingham, Birmingham, B15 2TT UK; 2 Birmingham Health Partners, Birmingham, B15 2TH UK; 3 Birmingham Community Health Care Trust, Birmingham, B7 4BN UK; 4 Ways for Wellbeing UK, Birmingham, B28 0ED UK; 5 Peak Health Coaching, Sheffield, S11 9RA UK; 6 Ashiana Community Project, Birmingham, B11 1LU UK; 7 Birmingham Voluntary Services Council, Birmingham, B1 2AJ UK; 8 Personalised Care Institute, London, SE1 7SJ. UK; 9 Northumbria University, Newcastle Upon Tyne, NE7 7XA UK; 10 United Kingdom and International Health Coaching Association, Tunbridge Wells, TN4 8BS UK; 11 NHS Birmingham, Black Country, and Solihull Integrated Care Board Cluster, Birmingham, B1 1TT UK; 12 School of Medicine, Shanghai Jiao Tong University, Shanghai, 200025 China

**Keywords:** Health and wellbeing coaching, Self-management, Underserved populations, Preventive health

## Abstract

**Background:**

The challenges in managing and treating the growing numbers with chronic ill health are often exacerbated in minoritized, isolated, and economically marginalized communities (“underserved populations”). One approach that has the potential to address persistent health inequalities in these populations is health and wellbeing coaching. Its ability to offer flexibility of timing and location, equitable communication, tailored content, and cultural congruency mean it is well-suited to engage with populations that mainstream care fails to adequately engage. To help understand the extent to which this is the case, this review will systematically explore the perspectives and experiences of health and wellbeing coaching amongst underserved populations, with the intention of identifying and exploring barriers and facilitators to its success and where possible suggesting practical strategies for improving its effectiveness and impact.

**Methods:**

Electronic databases including Medline/PubMed, CINAHL (Cumulative Index to Nursing and Allied Health Literature), Web of Science, PsycINFO, Cochrane Library and EMBASE were consulted. Included studies were qualitative, conducted in underserved populations with adults > 18yrs (i.e. rural areas, ethnic minorities, lower socioeconomic backgrounds, and other disadvantaged groups); where the interventions were categorised as health and wellbeing coaching delivered by trained coaches. The results are presented in an amended version of the Theoretical Framework of Acceptability.

**Results:**

A total of 483 studies were identified with nine included in the review from four different countries. In summary, participants were satisfied with health and wellbeing coaching noting the strength and value of their equitable relationship with the coach, how external responsibilities and economic pressures limited engagement; the benefits of demographically similar coaches that met linguistic and cultural preferences. The flexibility afforded by hybrid mHealth interventions was broadly welcomed, with the interventions tending to be successful in delivering notable change to mutually agreed goals.

**Conclusions:**

The techniques used in the identified studies reflect the strategies recommended in overcoming inequalities in access and utilisation of health and social care in a range of settings and the use of health and wellbeing coaching appears to offer viable and practical means of supporting lifestyle change and improving self-management.

**Trial registration:**

The review was registered on PROSPERO registration CRD42023472159.

**Supplementary Information:**

The online version contains supplementary material available at https://doi.org/10.1186/s12889-026-28440-8.

## Introduction

In high-income countries around the globe, the challenges presented by the growing prevalence of chronic conditions, including obesity, and mental ill health are exacerbated for those in underserved populations: for the purposes of this paper this collective phrase describes those who are economically deprived or disadvantaged [1], geographically isolated [2], or from racial and ethnic minorities, all of which are engaged less effectively by formal healthcare interventions or experiencing poorer outcomes and higher levels of service utilisation than other sections of the population [3–5]. These disparities are impacted by a range of socio-economic and cultural influences such as poor transport, housing, language barriers, low health literacy, and a lack of trust in mainstream healthcare organisations and providers [6–10].

**Fig. 1:**
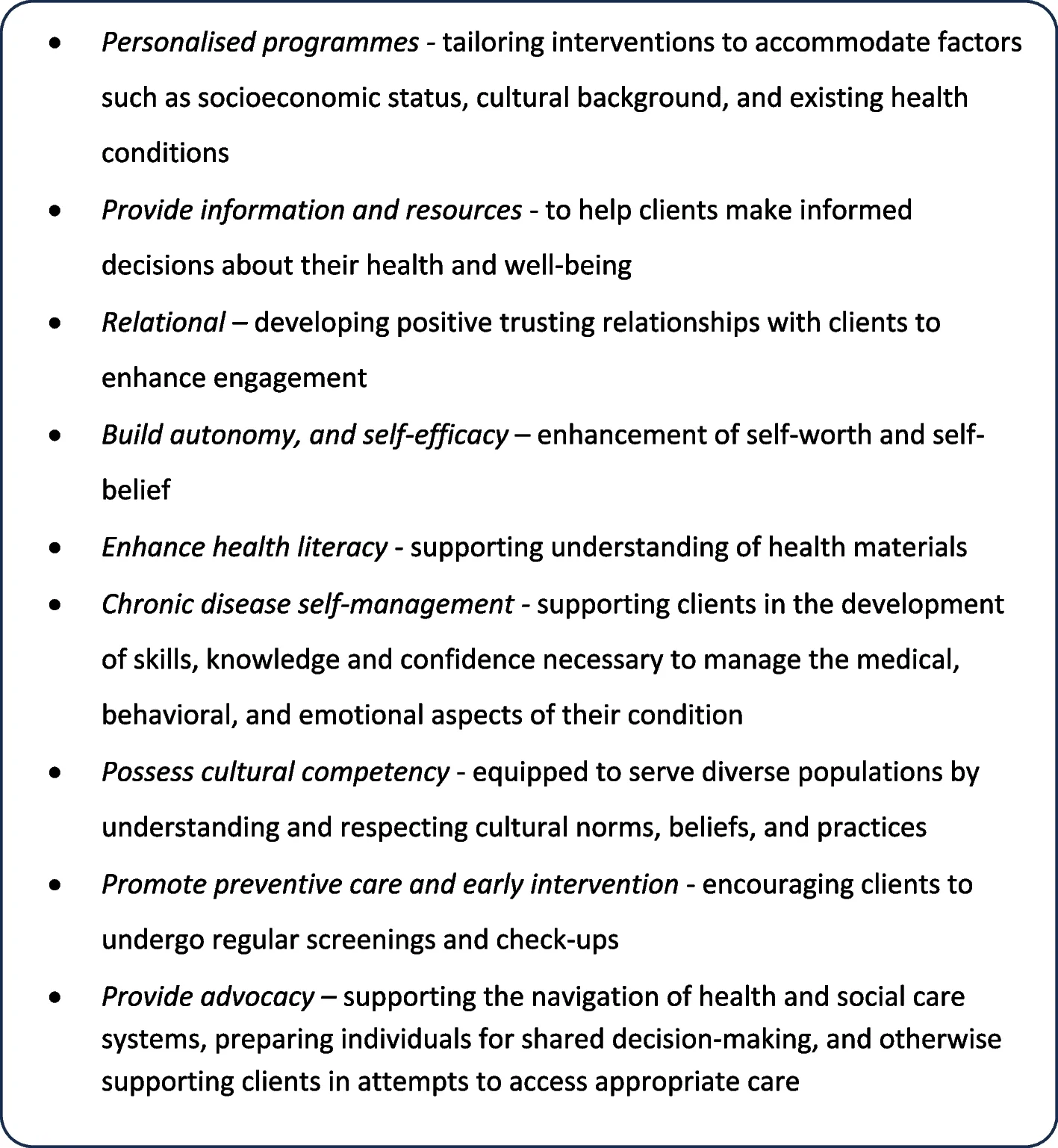
Key elements of health and wellbeing coaching

One approach that might address some of these barriers to access and reduce inequalities is health and wellbeing coaching (HWBC) [11]. This coaching is grounded in theories of patient-centred behaviour change, working with individuals to set mutually agreed and personalized goals, developing collaborative relationships, and helping them gain the ability, information and confidence necessary to reach these objectives [12–14]. The key elements of HWBC are further described in Fig. 1 [15]. The strategies used in HWBC such as equitable communication, tailored content, cultural congruency, and flexibility of timing and location [16–19], suggest it is particularly suited to engage with underserved populations helping to mitigate, if not overcome, many of the barriers to engagement with positive health-related behaviours [20, 21].

There is evidence from previous systematic reviews that where appropriately trained and deployed health and wellbeing coaches can improve a range of health and lifestyle factors including physical activity, weight loss [22], healthy eating [23], oral health promotion [24], mental health and well-being [25], as well as supporting self-management in a range of long term conditions [26]. However, what is less well understood is how effective HWBC is with underserved populations. If the benefits are to be consistently observed in these groups then a greater understanding of their experiences of HWBC is required to better shape the content, methods, and contextual support they need [18]. The aim of this work is to undertake a qualitative systematic review of the perspectives and experiences of HWBC amongst underserved populations to better understand its acceptability, identify and explore barriers and facilitators, and where possible suggest practical strategies for improving engagement and effectiveness.

## Methods

### Study design

We conducted a systematic review of qualitative and mixed method studies [27]. The search strategy combines search terms based on the SPIDER framework for qualitative research [28]. The results or our search are described in accordance with the Preferred Reporting Items for Systematic Reviews and Meta-Analyses (PRISMA) checklist [29]. The data are analysed using a directed content analysis [30, 31] to populate Sekhon’s Theoretical Framework of Acceptability (TFA) [32]. The review was registered on PROSPERO registration CRD42023472159.

### Theoretical lens

**Fig. 2:**
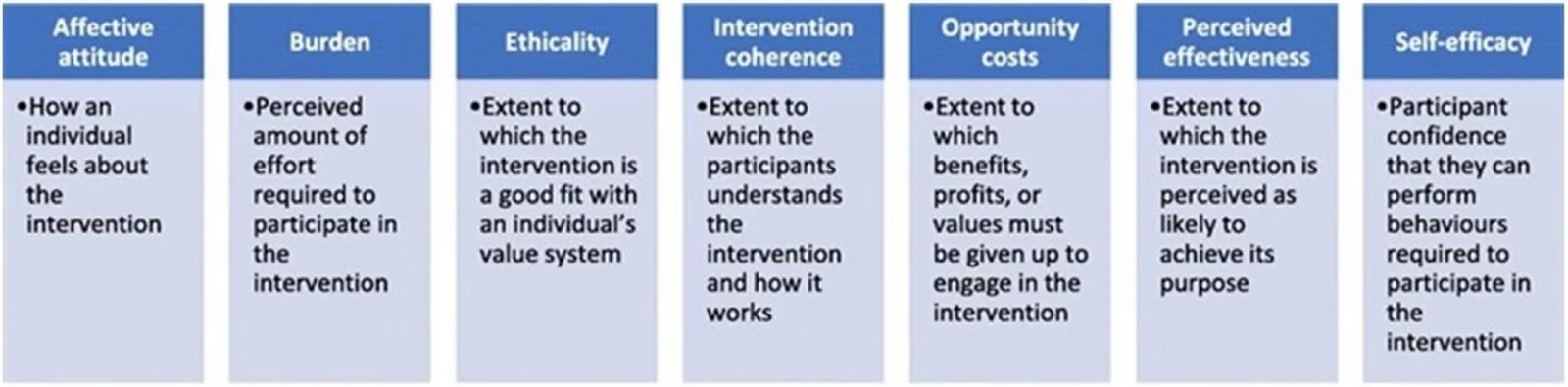
Summary of Sekhon’s theoretical framework of acceptability [32]

The TFA was developed by Sekhon et al. to explore acceptability of health care interventions from the perspectives of providers and patients [32]. It consists of seven domains: affective attitude, burden, ethicality, intervention coherence, opportunity costs, perceived effectiveness, and self-efficacy (see Fig. 2 for a brief definition of each domain). Together they describe whether those receiving (or delivering) an intervention consider it to be appropriate based on anticipated or experiential cognitive and emotional responses [32]. It has been successfully used in a range of settings and circumstances including understanding patient perspectives on the treatment of HIV [33], breast cancer [34], mental health [35], and diabetes [36].

### Search strategy

The search strategy was constructed in line with the SPIDER tool and described in Supplementary File 1. Electronic databases including Medline/PubMed, CINAHL (Cumulative Index to Nursing and Allied Health Literature), Web of Science, PsycINFO, Cochrane Library and EMBASE were consulted. Furthermore, the bibliographies of included papers were examined until no unreviewed studies surfaced, complemented by manual searches in Google Scholar (consisting of the first 50 results generated which were then screened for relevance).

### Study selection

The last author [ZZ] first performed title screening where studies with irrelevant titles were excluded. Studies with relevant titles proceeded to abstract screening, and those not meeting the criteria were excluded. The first author acted as second reviewer [IL] and conducted a full-text review of the remaining studies to assess their eligibility. Ultimately papers were included following consensual agreement between the two reviewers.

Studies which met the following criteria were included; Underserved populations (to confirm this includes economically deprived or disadvantaged (i.e. the condition in which individuals or households struggle to meet their basic needs [1]), geographically isolated (which describes the physical separation of places or territories restricting social behaviour and mobility) [2], or from racial and ethnic minorities, all of which are engaged less effectively by formal healthcare interventions or experiencing poorer outcomes and higher levels of service utilisation than other sections of the population [3–5, 37–39]); research conducted with adults (18 years and older); where interventions were categorised as health coaching or health and wellbeing coaching; studies containing qualitative data (e.g., interviews, focus groups, or mixed-methods); peer-reviewed articles published in English within the last 20 years (to reflect recent health system environments).

### Quality assessment and data extraction

The two authors, [IL and ZZ] independently assessed the quality of each included study. The Mixed Methods Assessment Tool (MMAT) [40] was used for qualitative studies and those studies of mixed-method design with qualitative components. The scores are reported within the table summarizing study characteristics. The first author IL extracted data including publication information, intervention and participant characteristics, and patient experiences (the study selection and data extraction templates can be found in Supplementary File 2.) Where studies employed mixed methods, quantitative data were also summarised to provide additional context. The data included in the analysis consisted of verbatim quotes from participants and the study authors’ summaries of findings.

### Data analysis

The data were analysed using a directed content analysis, populating the TFA using Elo and Kyngas’s open matrix approach that allowed for the inclusion of emerging constructs within existing domains [30, 31]. The process was collaborative, with IL and ZZ independently allocating data within the framework and with the final allocation consensually agreed by all authors.

## Results

### Characteristics of included studies

**Fig. 3:**
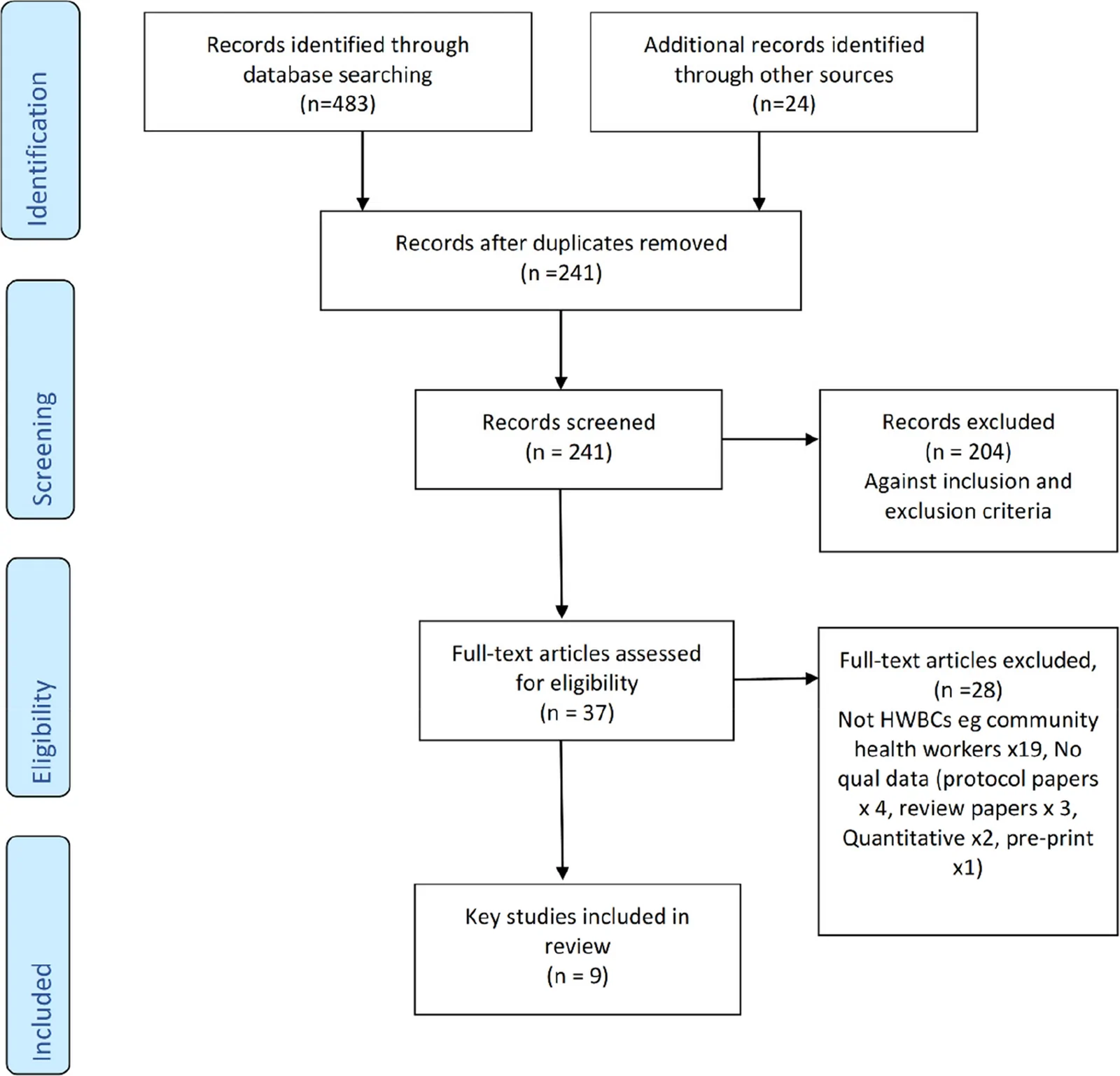
PRISMA flow diagram of the studies included in the review

The screening and study selection are outlined in the PRISMA (Preferred Reporting Items for Systematic Reviews and Meta-Analyses) flow diagram (Fig. 3). The final search was conducted January 31st, 2026. Studies were excluded because of duplication, not including interventions delivered by health and wellbeing coaches, or not being original peer-reviewed research. The PRISMA flowchart is shown in Fig. 3.

A total of nine studies were included. These consisted of two qualitative [41, 42], and seven mixed methods studies [43–49]. They were conducted in four different countries: six in the United States of America (USA) [41, 43, 45, 47–49], one in Canada [44], one in China [42], and one in the UK (and China) [46]. Their characteristics are further summarized in Table 1.

**Table 1:** Key c haracteristics and findings of included studies

Author (date)	Country	Study design (Quality)	HWBC intervention	Participants	Qualitative data collection	Outcome data (where applicable/available)	Summary of qualitative data
Boykins et al. 2025 [43]	USA	Mixed methods (High)	Provided individual and group health coaching Content focused on chronic illness management, exercise, or nutrition	Medically underserved clients (n = 1,355) Median age = 66.5 years, 93% Black/African American, 71% female	Semi-structured interviews (n = 14)	The majority (68.3%) participated in individual HWBC. Sixty-eight group sessions had an average of 4 participants (range 1–13)	Coaching can expand access to primary care and address social barriers to self-management in chronic conditions
Hohenadel J, et al. 2007 [44]	Canada	Mixed methods (Medium)	Specially trained coach navigators provided information and resources tailored to patients with cancer (part of a personal coaching programme involving health and social care providers)	Cancer patients identified with barriers of language, poverty, culture, social isolation, and literacy. (n = 63)	Semi-structured interviews (n = 11), Post intervention surveys (n = 27)	Survey data gathered across six domains: cancer information, practical support for living, emotional support, communication with health care professionals, practical health needs and support with friends and families	Participants reported being highly satisfied with the programme in both surveys and interviews describing positive implications for both physical and emotional health
Russell et al. 2018 [45]	USA	Mixed methods (Low)	A programme to address the social, health, and long-term disaster recovery needs (post-hurricane)	Community residents impacted by hurricane (n = 732)	Notes were taken during each encounter between providers and residents (n = unrecorded)	Referrals to medical and dental services (29%), city-issued identification cards (27%), and health insurance and other entitlements (23%)	Improvements in self-reported health, health care utilization, and confidence managing health issues
Steinman et al. 2023 [48]	USA	Mixed methods (High)	Part of a community-academic partnership to build capacity for evidence-based depression care	Older adults living in poverty with multiple chronic conditions and poor access to quality health care (n = 152)	Participant testimonials (n = 9)	Participants showed significant reductions in depression severity at 5 months (98% clinical response rate [mean (SD), 13.7 (3.9) drop in pre/post PHQ-9; *p* < 0.001]	Effective elements were reported. These included adaptability, staff buy-in, alignment with organization mission, ongoing reflection and evaluation during implementation
Schoenberg et al. 2021 [41]	USA	Qualitative (high)	Healthy lifestyle app supported by personalized health coaching, accelerometer and financial incentives	Rural population with lower rates of access to health and social services, internet and technology use, economic status, in four states in southeast USA (n = 54)	Focus groups (n = 4) Semi-structured interviews (n = 16)	N/A	Use of personal technology is feasible and desirable. Barriers included lack of healthy lifestyle awareness, habitual behaviour, and financial constraints
van Charante et al., 2024 [46]	UK	RCT (Medium)	Health coaches guide participants through mHealth platforms to manage dementia risk factors and assist them in using mHealth apps	Lower socio-economic status individuals with at least two risk factors for dementia (n = 1488) Age = 55–75 yrs Male (n = 601) and Female (n = 887) Intervention (n = 734) Control (n = 754)	Questionnaires and oral feedback to coaches	After a mean follow-up of 16 months (SD 2·5), the mean CAIDE score improved 0·16 points in the intervention group versus 0·01 in the control group (mean difference –0·16, 95% CI –0·29 to –0·03)	The mHealth intervention was considered appropriate and acceptable
Willard Grace et al. 2021 [49]	USA	Mixed methods (High)	Health coach provided COPD education, review correct inhaler use, counsel on smoking cessation, and facilitate other behaviour changes. Contact included in-person meetings, phone calls, and medical visits	COPD patients with varying levels of significant anxiety or depression, substance abuse, or homelessness (n = 20) Average age = 62 yrs Female = 30%	Semi-structured interviews with patients (n = 20)	13.7 interactions with health coach per patient Improved COPD management, symptom awareness, and behaviour change patients included those experiencing substance use or homelessness	Key elements of HWBC included the strength of the health coaching relationship and flexibility of the role
Yuan et al., 2022 [47]	USA	Mixed methods (Medium)	Health coach facilitator implementing a purposely designed mobile app Content includes education on core areas of integrative health, homework exercises, and evaluation activities	Patients using federally qualified community healthcare centre (n = 40) Female (n = 30) Average age 45.4 yrs Hispanic (n = 19)	User satisfaction surveys data from group sessions (n = 5) and individual sessions (n = 36)	Patients in the group setting had the highest rate of goal completion Significant (*p* <.05) changes in overall wellbeing, global physical health, BMI, vigorous physical activity, and eHealth literacy	Participants found the app helpful for behaviour change and health management
Zhang et al., 2025 [42]	China (High)	Qualitative (high)	Health coaches guide participants through mHealth platforms to manage dementia risk factors and assist them in using mHealth apps	Low socio-economic status and two or more dementia risk factors and involved in the PRODEMOS trial (n = 14)	Semi-structured interviews (n = 50) at 3, 12 and 18 months into the intervention	N/A	Remote coaching was convenient, and the app easy to use, although asynchronous communication with coach an issue

### Qualitative findings

The TFA was refined to bring greater clarity and concision to the presentation of the results: the domain ‘*Ethicality’* has been renamed *‘Cultural sensitivity’,* a phrase more precisely describing the domain and its constructs as represented in this review. Due to overlaps in the findings *‘Burden’* and ‘*Opportunity Costs’* were grouped to form one domain as were the related domains of *‘Intervention Effectiveness’* and *‘Self-efficacy’* resulting in five final domains and a total of 11 constructs definitions of both are contained in Table 2 alongside a summary of the findings within each as they relate to the successful delivery of HWBC. Below we explore the findings within each domain and construct, using example participant quotes (shown in italics).

**Table 2:** Mapping of domains, defined constructs and factors influencing success

Domain	Definition	Construct	Definition	Factors influencing success
1.Affective attitude	How participants feel about health and wellbeing coach -led interventions	1.1 Overall satisfaction with coach and intervention	Satisfaction with the concept and the coach	• Participants described overall satisfaction with and acceptability of HWBC [41, 43, 44, 46] • The importance of the client-coach relationship [41, 43, 44, 47–49]
		1.2 Client-coach communication	The communication strategies and approaches used by coaches	• Coaches spoke to clients as equals, flattening hierarchy [42, 49] • Informal non-clinical coaches can reduce client concerns around health provider competency [49]
2.Burden/opportunity costs	Perceived amount of effort required to participate and the benefits or values that must be given up enabling participation	2.1 Health implications	The impact of physical or mental health on ability and motivation to participate	• Health complications affected ability to participate [44, 49] • Personal concerns and awareness of risks of ill health were a motivating factor [47]
		2.2 Socio economic barriers	The time and resources required for intervention participation	• Ability to engage and participate was inhibited by care or work responsibilities [42, 49], Poverty [41, 43, 44], Social isolation [44], and food security [41, 43]
3.Cultural sensitivity	How well intervention aligns with individual beliefs and values	3.1 Cultural and community awareness of coach	Cultural and community influences on engagement with healthcare including health related customs and traditions	• Social networks, religious beliefs, and gendered roles [41, 48, 50] • Preferences for coaches who shared demographic characteristics or health experience [43] • Coaches understanding of gendered roles i.e. *machismo* and *familio* in Latino populations [48]
		3.2 Language	Delivery of the intervention in multiple languages and tailored phraseology	• Communication was supported by the use of preferred languages [48] • There was a preference for simple but not patronising explanations [41]
4.Programme coherence	How far participants understand HWBC interventions and their construction	4.1 Content, methods and materials	Educational content, and strategies	• Useful information on managing specific conditions, specifically cancer [44], Depression [48], Dementia [42, 46], COPD [49], Cardiovascular risk [50] and chronic illness self-management [43] • Participants welcomed broader learning around multiple aspects of health, lifestyle, and social support [41, 42, 45–47] • Setting mutually agreed goals was effective [41–49]
			Communication modes	• There was appreciation for the flexibility of telephone and videoconferencing [47, 49] and the use of mHealth apps [42] data [41, 46, 47] • Issues with digital literacy [42] and impersonal and asynchronous communication [47]
		4.2 Structure	The use of group and/or one-to-one sessions and their frequency and duration	• Group sessions were noted for the support gained from peers [42] • Individual sessions favoured for their personalised support [41, 44, 45, 48–50] • Greater frequency of contact supported adherence [49, 50] • Lengthier conversations with coaches compared favourably to shorter clinical consultations [42]
5.Effectiveness and self-efficacy	The degree to which the intervention achieved and sustained its aims	5.1 Changed health behaviours	Impact of interventions on a range of self-management and lifestyle behaviours	• Substantive behaviour changes made with the support of their coach [41, 43–48]
		5.2 Increased self-efficacy and sustained change	The confidence and improved self-esteem when independently managing health behaviours	• HWBC increased self- confidence in managing health and well-being [43, 45, 47, 49] • The influence of the coach stayed with them after the programme ended [42, 49]

#### 1. Affective attitude

##### 1.1 Overall satisfaction with intervention and coach

Studies reported participants’ overall satisfaction with HWBC [41, 46], noting that they met their broad requirements for support and signposting considered necessary to improve a range of health behaviours [42–44]. A key element appeared to be the strength of the relationship developed with the coach, with participants describing its foundation in mutual trust and respect [44, 47] and a source of meaningful emotional support [41, 43, 47–49]. As one participant described:

“So, she was instrumental in…just keeping a good attitude, I guess….[I] could always call her up or text her - or something - with a question, and she always came back with an answer, and it made me smile or feel good.” [49]

For some, the closeness of the relationship also became a motivating factor for adherence to the program, with participants in one study describing how they felt compelled to commit to the intervention to prevent disappointing the coach:

"When you do it by yourself, most people will fail. But, if they have a coach… that accountability is there where you are not just letting yourself down…you don’t let your friend down." [41]

##### 1.2 Equitable communication

Coaches were recognized for the informal and more natural communication style which meant participants found it easier to discuss their issues and challenges [43, 48, 49], including in one study potentially sensitive conditions such as depression [48]. As one participant described:

"…The other thing is that a health coach can be someone who has medical training but they're not a doctor. People are intimidated by doctors. They don't see a doctor as somebody they can just sit down and have a cup of coffee and talk about their health." [49]

#### 2. Burden/opportunity costs

##### 2.1 Health implications

The impact of physical or mental health on the ability to participate was described, including the effects of competing co-morbidities [44, 49]. However, concerns over health were also a significant motivating factor, particularly when there was personal experience of ill-health [42, 44]. As a participant from an intervention attempting to decrease the risk of dementia explained:

“I take my health more seriously now. Last year, my brother died suddenly at the age of 66, which was a big blow for me. So, I thought that we should be kinder and more responsible to ourselves.” [47]

##### 2.2 Socio-economic barriers

Participants reported a number of barriers to participation including poverty [41, 43, 44], social isolation [44], and a lack of food security [41, 43]. For example, the economic challenges of pursuing a healthier diet were summarized by a participant in rural USA:

"…the health issues that we have here in eastern Kentucky are a byproduct of financial burdens. The old joke is nutrition is more expensive than drugs". [41]

Participants also described how caring responsibilities [49] and work commitments [42] also impacted their ability to participate:

"I am not fully retired and still working, so my time available for health management and the app usage is still relatively limited because I rarely have a chance to rest when I am busy with my work/routines." [42]

#### 3. Cultural sensitivity

##### 3.1 Cultural and community awareness

Participants described how the influence of their religious and cultural roles and activities affected their engagement with health coaching [41, 48]. For example, a participant in a study in rural USA noted how their church-going peers were becoming increasingly aware of the influence of lifestyle on chronic disease:

"People are getting more interested in physical activity and want to do better. Being African American, I know I have many friends who are concerned with blood pressure and diabetes" [41]

The importance of coaches understanding the customs, traditions, and cultural traditions of their clients was described by participants [43, 48]. This included in one study how their experience was improved by providers who shared their gender, race, and cultural background [43]. In another conducted in Latino populations the benefits of coaches being aware of the inhibitive influence of cultural values of “*familismo”* (commitment and loyalty to family expected of mothers) meant they were able to provide explicit encouragement to put their needs before their adult children [48].

##### 3.2 Language

Conversations around behavioural change can be complex and nuanced and so where possible were conducted in participants’ preferred language [48]. Health literacy can also vary in underserved populations, and though it was important this was reflected in the vocabulary used, it was felt important that coaches didn’t appear to talk down to the participant:

“I think you have to watch with people in this area about how you present things as well. They are easily made to feel like they didn’t know ‘that’ so they must be dumb" [41].

#### 4. Intervention coherence

##### 4.1 Content, methods and materials

Interventions included practical information on health and care, lifestyle factors, and social support [41, 42, 45–47], with some including more specific information on long-term conditions specifically cancer [44], depression [48], dementia [42, 46], chronic obstructive pulmonary disease (COPD) [49], and chronic illness self-management [43]. All interventions involved setting mutually agreed goals [41–49], six used hybrid in-person/digital methods incorporating face-to-face sessions, alongside telephone and videoconferencing [47, 49] and/or a HWBC app [41, 42, 46, 47]. These apps were noted for their ability to offer a more flexible and accessible alternative to accessing information [41, 46, 47]. As one participant explained:

“The advantage of the project is that we can communicate about these things online… and the app and coach always give me advice…” [47]

There were however some concerns around the use of the app: some felt communication was impersonal and were unhappy with the length of the delay in receiving a response from their coach [47]. Meanwhile, elderly participants felt the interface could be better designed to upload data directly from worn technologies to reduce the work of manually entering measurements. As one participant described:

“The app should be designed to be more concise, with fewer entering procedures” [42].

##### 4.2 Structure

The HWBC interventions, included individual [41, 44, 45, 48, 49] and/or group sessions [42, 43, 46, 47]. The benefits of both were described, with participants that used individual sessions appreciating their personalized approach and the targeted encouragement:

“A lot of people are too scared to start. By getting the one-on-one alone time it will help people to not give up and they will be more likely to follow through”. [41]

There were also advantages reported specific to group sessions including emotional support provided by “classmates.” [43] and the power of encouragement delivered by peers:

“I believe we can encourage each other to exercise more. It's like, we could be each other's reminder, you know?“ [42]

None of the studies included in the review provided specific information on the length of each HWBC session or their number. However, participants in one study noted that coaches had more time to listen than busy clinical staff and that the frequency of contact was instrumental to their adherence. Leading one participant to comment:

“To be honest, I probably couldn't stick with it if the coach didn’t regularly supervise me.” [42].

#### 5. Effectiveness and self-efficacy

##### 5.1 Changed health behaviours

Participants described how interventions improved their health and care behaviours in a number of ways, including facilitating better engagement with care and other services [43–45]; specific clinical outcomes [43, 46, 49]; broad improvements in physical and mental health and wellbeing [44, 45, 47, 48]; and a number of related lifestyle factors [47, 49]. As one participant with COPD described:

"I quit smoking and quit drinking and everything. I do exercises every day. I come down to [name of] Park, and I walk two laps every day to build up my lungs and my breathing." [49]

However, not all felt their ability to change was due to the HWBC intervention, with one participant preferring to credit their individual willpower:

Interviewer: Do you think there's anything a health coach could do to help you quit?

Patient: No. I just have to make up my mind to quit.

Interviewer: All up to you?

Patient: Yeah, it's up to me. [49]

##### 5.2 Increased self-efficacy and sustained change

Participants described how their coaches support grew confidence in their own ability to successfully manage a range of health and care related lifestyle behaviours [43, 45, 47, 49]. In one study a participant with COPD described how these changes were sustained after the end of the intervention through the strategies provided by their coach:

“She was always asking questions… "Oh, before you have a drink, what are you thinking about?" That was like, "Whoa." But it was good because now when I want to have a cigarette or get an extra beer, I always say, "Why do I want to do this?" … I think about it, and most of the time when I start thinking about it, then I don't do it”. [49]

## Discussion

### General summary

Health and wellbeing coaching offers the potential to overcome some of the persistent socio-cultural barriers that prevent underserved populations engaging in positive health and wellbeing-related behaviours. The review confirmed much of this potential and the TFA proved a useful means of structuring our findings. In summary by domain: Participants described, their satisfaction with the HWBC intervention, noting the strength and value of the equitable relationship developed with their coach (*Affective attitude)*; how participation was inhibited by work and carer responsibilities and economic constraints (*Burden/opportunity costs)*, the benefits of demographically similar coaches and their ability to understand and address cultural pressures on lifestyle change (*Cultural sensitivity)*; the value of a range of practical lifestyle and social support elements, group and individual sessions, and hybrid services offering mHealth apps in conjunction with in-person sessions (*Intervention coherence)*; finally, participants reported fundamental and lasting changes in a range of health and wellbeing-related behaviours (*Effectiveness and self-efficacy)*.

### Strengths and limitations

The review collated the valuable perspectives of underserved populations participating in HWBC of various design and purpose. The TFA’s comprehensive structure added to the review’s methodological rigour which followed best practice in systematic review methods throughout [51]. Although the overall quality of the studies was high, the number of studies identified was small and their design heterogenous. One study of low quality was included because of the desire to explore all available evidence [52]. It is also worth noting that attritional bias may also impact the findings with the likelihood that only those most engaged in the intervention completed surveys or interviews [53]. Despite these limitations, the findings suggest that the methods and strategies used in HWBC were not only broadly acceptable to clients in underserved populations but also preferred over medically oriented and clinically delivered alternatives. In doing so they reflect existing evidence that underserved patients better engage in health and social care that understands their socio-cultural attitudes and status, health literacy, and self-efficacy, barriers that otherwise negatively impact their engagement and adherence to positive health-related behaviours [6, 8, 54].

### Specific findings

#### Affective attitude

Participants in multiple studies described the importance of the open and collaborative relationship developed with their coach [41, 43, 44, 47–49]. This reflects previous evidence that highlights how the longitudinal relationships between coach and client can develop trust and understanding and are a foundational element of successful HWBC [14, 55, 56]. Its long been recommended that clinicians develop similarly equitable and constructive relationships to maximize their therapeutic alliance with the patient [57, 58], yet attempts to share clinical decision making have failed to gain traction [59, 60] perhaps offering further explanation as to why participants preferred to speak with coaches instead of clinicians [42, 49].

#### Burden and opportunity costs

The review described a range of familiar barriers to engaging with the HWBC intervention including ill health [44, 49], care and work responsibilities [42, 49], and economic and social disadvantages, including the cost of food [41, 43, 44]. The prohibitive expense of “healthy” eating has figured before in attempts to encourage healthier lifestyles [61], and there is evidence that the use of food coupons or prescriptions might be helpful in future iterations [62, 63].

One motivating factor which emerged in the review was the recent personal experience of ill-health amongst family or relatives [42]. However, evidence of the degree to which personal motivation affects the initiation or maintenance of behaviour change is unclear [64, 65], particularly in underserved populations [66, 67]. Related to this was the attitude of one participant who felt the ability to change was solely due to their will power failing to consider the impact of external factors [49], a seemingly common misperception in lifestyle change [68].

#### Cultural sensitivity

There are a range of culturally specific influences that are acknowledged as impacting healthy behaviours in minoritized populations including gender, religion, and custom [7], and its widely recognised that any support for behaviour change accommodates these socio-cultural traits [69, 70]. In Latino populations these cultural factors include strongly gendered roles for men and mothers [71, 72] and the review noted the benefit of their health coach understanding these challenges [48]. Another way familial pressures might be addressed is by delivering family-level interventions that reduce the pressure placed on mothers otherwise confronted with pursuing lifestyle changes alone [73].

The importance of using a patient’s preferred language is also widely understood across multiple health and care services and support [74]. The provision of coaching and supporting materials in Spanish for Latino populations described in the review [48] follows previous recommendations for delivering self-management and behavioural interventions in the preferred language of recipients [75], including the use of Spanish in HWBC in Latin populations [76]. However, providing content in a familiar language does not itself overcome issues of health literacy which can predominate in underserved populations [77, 78]. One study identified in the review noted that care should be taken to provide support in plain language that is understandable without being patronising [41]. The importance of tailoring health communication and adapting content to meet individual needs and context is well-established [77] and one approach that has previously improved comprehension of health-related information in a range of settings is co-production; a process that consensually generates content with target populations and other stakeholders [79, 80].

#### Intervention coherence

Interventions in the review included information targeting chronic illness [42–44, 46, 48–50]. This is reflective of the increasing use of HWBC in self-management [81–85], with statistically significant improvements being reported in a range of physical and mental health indictors in the broader population [86]. The interventions in the review also offered information around multiple lifestyle factors [41, 42, 45–47, 87], reflecting the varied roles of coaches as cultural mediators, liaison workers, navigators, and advocates [88]. Both navigation and advocation are particularly critical for underserved populations where they have been linked to a reduction in health inequalities [89, 90].

Multiple studies described setting mutually agreed goals [41–49, 87], a personalised approach central to HWBC and that has produced positive effects in a range of settings and conditions [91–94]. This personalised approach understands how individuals more carefully consider a message where it is targeted and personally relevant [95, 96]. Although the strategy is effective across the broader population, there is increasing evidence that it is particularly helpful in underserved populations where it can help accommodate sometimes challenging individual contexts [54, 97].

A number of studies reported a positive reaction to the increased accessibility provided by hybrid mHealth facilitated interventions [41, 42, 46, 47], reflecting the growing use of complementary digital technologies in HWBC [98]. Used appropriately there is evidence in other elements of the population that these apps can improve communication and enable all involved to evaluate fidelity to the intervention, monitor progress and adverse events, and identify areas for improvement [99]. Latterly this includes the rise of AI-based technology in multiple apps that also appear to support health behaviour change [100, 101].

Implementing any digital health tool is subject to issues of connectivity, affordability, and digital literacy, and these factors have so far limited their uptake in underserved populations [102, 103]. Digital literacy was also an issue raised in the review, manifest in concerns around the digital interface and the onerous nature of uploading data [42, 47]. The latter might be overcome by better integrating wearable technologies [104], with examples now emerging of their favourable use in HWBC interventions [105].

Identified studies included various combinations of individual [41, 44, 45, 48–50] and group sessions [42, 43, 46, 47], with debates over the advantage and efficacy of each ongoing for decades, with little clear evidence in favour of either [106, 107]. However, group sessions offer the potential for cost savings and greater reach [108]. The presence of peers can also provide additional motivation as observed in the review [42] and reflected in previous evidence that indicated witnessing similar individuals making positive changes can boost self-efficacy and empowerment [109–111].

#### Effectiveness and self-efficacy

The combined characteristics unique to HWBC can support self-efficacy and meaningful behavioural change in populations that mainstream health services often fail to engage [20, 21, 112]. This ability was further evidenced by this review where the HWBC intervention increased self-efficacy [43, 45, 47, 49] and supported positive change in various health-related behaviours [41, 43–48, 87]. Participants in two studies described how the influence of the coach and their messaging remained beyond the intervention [42, 49]. What was less clear was how long change was maintained, and though there is some evidence it can be sustained it is frequently underreported in HWBC studies [113, 114]. It has been recommended that longevity can be supported in chronic conditions where the methodologies employed in HWBC are embedded across the care team who adopt the same language and strategies [112]. This integration presents its own issues of compatibility, and more work is required to embed HWBC into existing clinical models of care to overcome challenges of organizational understanding and support [15, 115].

## Conclusions

This review offers further evidence of how the unique combination of characteristics of HWBC i.e. flexibility, consensual goal setting, and equitable relationships can mitigate challenges of culture, health literacy and logistical restrictions experienced in underserved populations. Greater consistency in study design and reporting would strengthen the evidence base and help inform future HWBC programs for underserved populations.

## Supplementary Information

Supplementary Material 1.

Supplementary Material 2.

## Data Availability

No datasets were generated or analysed during the current study.

## Abbreviations

CINAHL: Cumulative Index to Nursing and Allied Health Literature
HWBC: Health and Wellbeing Coaching
MMAT: The Mixed Methods Assessment Tool
PRISMA: Preferred Reporting Items for Systematic Reviews and Meta-Analyses
TFA: Theoretical Framework of Acceptability
UK: United Kingdom
USA: United States of America
