# Supplementary material for: Understanding the influence and acceptability of health and wellbeing coaching in underserved populations: a qualitative systematic review
Source: BMC Public Health. 2026 Aug 5;26:3149. doi: 10.1186/s12889-026-28440-8 (PMC13726219; doi:10.1186/s12889-026-28440-8)
Supplement: Supplementary file 1 [file 12889_2026_28440_MOESM1_ESM.docx]

**Supplementary File 1**

*Understanding the influence and acceptability of health and wellbeing coaching in underserved populations: A qualitative systematic review*

Litchfield et al

**ST1: Sample of search term strategy using SPIDER (Eggleston, 2021)**

| **Sample (S)** | **Phenomenon of Interest (PI)** | **Design (D)** | **Evaluation (E)** | **Research type (R)** |
| --- | --- | --- | --- | --- |
| Adults (≥18)  underserved communities* OR ethnic minorities* OR economically disadvantaged* OR rural populations* | Health coaching* or Health and well-being coaching* | questionnaire* OR survey* OR interview* OR focus group* | view* OR experience* OR opinion* OR attitude* OR perception* OR belief* OR feel* OR know* OR understand* OR challenges | Qualitative* OR Mixed method*ds |

**ST2**

**Table S2: Eligibility criteria**

|  | **Inclusion criteria** | **Exclusion criteria** |
| --- | --- | --- |
| **Sample population** | - Adults in underserved populations | - Under 18 - Those populations described as being in privileged, advantaged, well-served, or affluent populations |
| **Phenomena of Interest** | - The use of trained health and well-being coaches or structured coaching | - Any other health or well being offer from peer supporters, lifestyle coaches, wellness coaches or those without training in health and well-being coaching methods and approaches |
| **Design of study** | - All theoretical approaches - Data collection method either focus groups or interviews, or survey data containing free text or all three - Any analysis methodology | - Quantitative designs |
| **Evaluation** | - Experiences of participants providers - Perspectives, experiences and attitude toward Health and well being coaches |  |
| **Research Type** | - Qualitative studies - Mixed Methods | - Quantitative studies |

**Search terms (MEDLINE)**

*Underserved populations*

("disadvantage"[All Fields] OR "disadvantageous"[All Fields] OR "disadvantageously"[All Fields] OR "disadvantages"[All Fields] OR "disadvantaging"[All Fields] OR "vulnerable populations"[MeSH Terms] OR ("vulnerable"[All Fields] AND "populations"[All Fields]) OR "vulnerable populations"[All Fields] OR "disadvantaged"[All Fields] OR ("underserved"[All Fields] OR "underserviced"[All Fields] OR "underservicing"[All Fields]) OR (("economical"[All Fields] OR "economics"[MeSH Terms] OR "economics"[All Fields] OR "economic"[All Fields] OR "economically"[All Fields] OR "economics"[MeSH Subheading] OR "economization"[All Fields] OR "economize"[All Fields] OR "economized"[All Fields] OR "economizes"[All Fields] OR "economizing"[All Fields]) AND ("deprival"[All Fields] OR "deprivation"[All Fields] OR "deprivations"[All Fields] OR "deprive"[All Fields] OR "deprived"[All Fields] OR "deprives"[All Fields] OR "depriving"[All Fields])) OR ("low"[All Fields] AND ("social class"[MeSH Terms] OR ("social"[All Fields] AND "class"[All Fields]) OR "social class"[All Fields] OR ("socio"[All Fields] AND "economic"[All Fields] AND "status"[All Fields]) OR "socio economic status"[All Fields]))) AND ("ethnical"[All Fields] OR "ethnically"[All Fields] OR "ethnicities"[All Fields] OR "ethnicity"[MeSH Terms] OR "ethnicity"[All Fields] OR "ethnic"[All Fields] OR "ethnics"[All Fields] OR "ethnology"[MeSH Subheading] OR "ethnology"[All Fields] OR "ethnology"[MeSH Terms]))) AND (y_5[Filter])

*Health coaching*

(("health"[MeSH Terms] OR "health"[All Fields] OR "health s"[All Fields] OR "healthful"[All Fields] OR "healthfulness"[All Fields] OR "healths"[All Fields]) AND ("coach"[All Fields] OR "coach s"[All Fields] OR "coached"[All Fields] OR "coaches"[All Fields] OR "mentoring"[MeSH Terms] OR "mentoring"[All Fields] OR "coaching"[All Fields]))

*Qualitative research*

("Qualitative Research"[MeSH] OR "qualitative" OR "interview" OR "focus group" OR "grounded theory")

*Mixed methods*

("mixed"[All Fields] OR "mixes"[All Fields] OR "mixing"[All Fields] OR "mixings"[All Fields]) AND ("method s"[All Fields] OR "methods"[MeSH Terms] OR "methods"[All Fields] OR "method"[All Fields] OR "methods"[MeSH Subheading])
