# Supplementary material for: Understanding the influence and acceptability of health and wellbeing coaching in underserved populations: a qualitative systematic review
Source: BMC Public Health. 2026 Aug 5;26:3149. doi: 10.1186/s12889-026-28440-8 (PMC13726219; doi:10.1186/s12889-026-28440-8)
Supplement: Supplementary file 2 [file 12889_2026_28440_MOESM2_ESM.docx]

**Supplementary File 2 Study selection + data extraction forms**

**Table S2a: Study Selection Form**

| Study information | Study ID |  |  |  |
| --- | --- | --- | --- | --- |
|  | Author |  |  |  |
|  | Year |  |  |  |
|  | Title |  |  |  |
| Inclusion Criteria  (Y/N) | Is the study either qualitative or mixed methods (containing qualitative elements)? |  |  |  |
|  | Is the study collecting a sample from underserved populations? |  |  |  |
|  | Are the GPs in primary care? |  |  |  |
|  | Is the study either qualitative or mixed methods (containing qualitative element)? |  |  |  |
|  | Does the study report experiences of health coaching from underserved populations |  |  |  |
| Exclusion Criteria  (Y/N) | Does the study not contain qualitative elements? |  |  |  |
|  | Are they from a population that is not otherwise defined as underserved? |  |  |  |
|  | Does the study not explore the experience of health coaching? |  |  |  |
|  | Include or exclude |  |  |  |
|  | Reason for exclusion |  |  |  |

**Table S2b: Data extraction form**

| **Study information** | Study ID |  |  |  |
| --- | --- | --- | --- | --- |
|  | Author |  |  |  |
|  | Year |  |  |  |
|  | Title |  |  |  |
| **Study characteristics** | Country of study |  |  |  |
|  | Setting |  |  |  |
|  | Participant interview or free text survey |  |  |  |
|  | Sample number in the study |  |  |  |
|  | Data collection method? |  |  |  |
|  | Specific focus of Health and wellbeing coaching? |  |  |  |
|  | Study analysis method |  |  |  |
|  | Underserved populations perspectives? |  |  |  |
| **Aims** | Study aims /purpose |  |  |  |
| **Study outputs** | Main themes |  |  |  |
|  | Recommendation/application |  |  |  |
